# Assessment System for Predicting Maximal Safe Range for Heel Height by Using Force-Sensing Resistor Sensors and Regression Models

**DOI:** 10.3390/s22093442

**Published:** 2022-04-30

**Authors:** Yi-Ting Hwang, Si-Huei Lee, Bor-Shing Lin

**Affiliations:** 1 Department of Statistics, National Taipei University, New Taipei City 237303, Taiwan; hwangyt@gm.ntpu.edu.tw; 2 Department of Physical Medicine and Rehabilitation, Taipei Veterans General Hospital, Taipei 112, Taiwan; 3 Faculty of Medicine, National Yang Ming Chiao Tung University, Taipei 112, Taiwan; 4 Department of Computer Science and Information Engineering, National Taipei University, New Taipei City 237303, Taiwan

**Keywords:** high-heeled shoes, plantar pressure, force sensing resistor sensors, regression model, heel height

## Abstract

Women often wear high-heeled shoes for professional or esthetic reasons. However, high-heeled shoes can cause discomfort and injury and can change the body’s center of gravity when maintaining balance. This study developed an assessment system for predicting the maximal safe range for heel height by recording the plantar pressure of participants’ feet by using force-sensing resistor (FSR) sensors and conducting analyses using regression models. Specifically, 100 young healthy women stood on an adjustable platform while physicians estimated the maximal safe height of high-heeled shoes. The collected FSR data combined with and without personal features were analyzed using regression models. The experimental results showed that the regression model based on the pressure data for the right foot had better predictive power than that based on data for the left foot, regardless of the module. The model with two heights had higher predictive power than that with a single height. Furthermore, adding personal features under the condition of two heights afforded the best predictive effect. These results can help wearers choose maximal safe high-heeled shoes to reduce injuries to the bones and lower limbs.

## 1. Introduction

In modern society, wearing high-heeled shoes is popular among women. Women wear such shoes despite their adverse effects such as foot muscle soreness and back heel wear. A survey estimated that 62% of female American adults regularly wear shoes with a heel taller than 2 inches [1]. The long-term costs of wearing high-heeled shoes to the body are considerable. Years of wearing high-heeled shoes can not only cause various foot deformities and knee, ankle, and back problems but also alter the anatomy of the calf muscles and tendons [2].

High heels disturb the natural function and position of the ankle joint by forcing the foot into plantar flexion. In 2003, Esenyel et al. performed quantitative measurements of the lower-extremity joint function, including the angular motion, muscular moment, power, and work, and found that wearing high-heeled shoes may cause strain and discomfort to the lower extremities [3]. Electromyography tests of participants wearing shoes with heels of different heights revealed increased peak pressure in the forefoot and a change in the load distribution on the midfoot region while wearing shoes with higher heels [4].

In 2014, a study performed kinematic measurements and revealed that walking in high-heeled shoes significantly reduces ankle muscle movement and balance control during the transition in gait [5]. In 2010, another study revealed changes in the estimated length and cross-sectional area of the gastrocnemius medialis and Achilles tendon [6]. Studies investigating the long-term use of high-heeled shoes found changes in the neuromechanics of walking and greater strain on the muscles and tendons of the lower legs, which can lead to musculoskeletal disorders [7,8]. In particular, the data showed that the effects of high-heeled shoes are not localized to the foot but extend from the lower limbs to the spine.

Wearing high-heeled shoes leads to excessive plantar flexion during walking and accelerated muscle fatigue, which can affect foot stability and may cause ankle sprains and falls. A physician can determine whether a shoe’s height is safe for a user by observing the heel deformation and the change in both the medial and the lateral longitudinal arches. Previous studies have examined the effects of high-heeled shoes on balance and locomotion; however, few have discussed the maximal safe range of heel height for individuals. It can be found from past research that when the height of high heels is higher, the pressure on the forefoot will increase and the pressure on the hindfoot will decrease [9,10,11].

When wearing high heels, proper foot functioning requires adequate mechanical control over the tarsus of the foot, especially in the calcaneus and talus, which make up the subtalar joint that controls pronation and supination of the foot [11]. Thus, we can attempt to find the relationship between the changes in the height of high heels, the changes in plantar pressure, and the degree of misalignment of calcaneus and talus, that is, the appropriate height of high heels can be predicted through plantar pressure. In 2021, an innovative artificial intelligence (AI)-based system was proposed to evaluate the limit of heel height for female wearers [12]. In that study, in addition to the images collected from two cameras, values collected using force-sensing resistor (FSR) sensors on the soles of the feet were converted into images and then used AI to train and predict the limit of heel height. However, this method requires a high-performance computer with a graphics processing unit to produce the AI model, and determining which FSR sensors on the sole of the foot have decisive influence is impossible. In this light, the present study investigated an assessment method for predicting the maximal safe range of heel height only by recording the plantar pressure of a participant’s feet by using FSR sensors and the personal basic characteristics. To reduce the burden of tasks for the measurement, this study analyzed different heights to determine which FSR sensors most influence the heel height. This assessment method was based on the multiple regression model and an explicit formulation was provided. The maximal safe heel height can be determined by measuring two extremes of plantar pressures and this finding can be easily implemented in practice.

## 2. Methods

### 2.1. Study Population

In this study, the experimental procedure was approved by the Institutional Review Board of En Chu Kong Hospital, New Taipei City, Taiwan (No. ECKIRB1090404). In total, 100 healthy female participants who were familiarized with the experimental protocol and who provided informed consent before participation were enrolled in this study. The inclusion criteria were (1) age of 20–35 years; (2) no congenital scoliosis; (3) no calluses on the soles of the feet; (4) no history of serious lower limb injuries; (5) arch rate of 11–14%; (6) hallux valgus angle (HVA) ≤ 20°; (7) quintus varus angle (QVA) ≤ 20°; and (8) calcaneus varus or valgus angle (CVA) of rearfoot ≤ 4°.

The variables for criteria (5)–(8) were defined as follows: the arch rate was calculated as the measured height divided by the foot length and multiplied by 100% [13]. Here, the height refers to the height measured from the most convex point of the navicular bone to the ground while the participant was fully weight-bearing. The HVA is defined as the angle of intersection between the long axes of the first metatarsal bone and the proximal phalanx of the big toe [14]. The angle of intersection of the two lines is measured. The QVA is defined as the angle of intersection between the long axes of the fifth metatarsal and the proximal phalanx of the little toe [14]. CVA is defined as the angle between the bisection of the Achilles tendon and the midpoint of the calcaneus as measured when the participant stands on bare feet [14].

The additional variables collected in this study were the foot length, forefoot width, and rearfoot width. For measuring these variables, each participant placed her foot on a paper, and the researcher traced around the foot with a pen. The foot length was measured from the tip of the longest toe to the posterior aspect of the heel. The forefoot width was measured from the widest point of the first and fifth metatarsophalangeal joints. The rearfoot width was measured at 30% of the total length from the posterior heel to the forefoot bisection.

### 2.2. Measurement Platform

**Figure 1:**
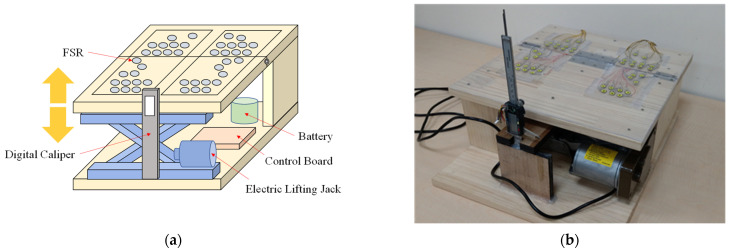
(**a**) Block diagram of the measurement platform; (**b**) Photograph of the measurement platform.

A measurement platform was needed to make the heel-rise automatically such that the physician could perform diagnoses. Further, this platform needed to incorporate plantar pressure sensors to record the plantar pressure of the participants’ feet. The platform consisted of five major components: FSR sensors, a control board, an electric lifting jack, a digital caliper, and a battery, as shown in Figure 1a. These were installed in a box with a length, width, and height of 40, 40, and 18.6 cm, respectively, as shown in Figure 1b. A total of 42 FSR sensors (FlexiForce A301, Tekscan, Boston, MA, USA) were installed in this system to detect the plantar pressure of both feet [15]. The characteristic curve of each FSR sensor is different, so we measured each FSR sensor and established its pressure–resistance characteristic curve. When developing the program, the characteristic curve formulas of these 42 FSR sensors were written into the program, and a reliable digital force gauge (DS-50, DESIK Instruments Group Limited, Karlsruhe, Germany) was used to verify its accuracy [16]. Therefore, calibration is not required before data collection. Specifically, 21 sensors each were installed for the left and the right foot, and they were mainly distributed on the forefoot and heel. The FSR sensors send data to the control board sequentially through two multiplexers (CD74HC4067, Texas Instruments, Dallas, TX, USA). The control board includes a TTGO module, DC-DC step-down power supply module (LM2596S), and H-bridge (BTS7960). The TTGO module collects the data from the 42 FSR sensors and a digital caliper and accordingly controls the lifting of the electric lifting jack. The collected data are transferred to the computer via a WiFi module in the TTGO module and saved as a CSV file. When users want to adjust the heel height of the measurement platform, the computer transmits the desired target height to the TTGO module over WiFi; then, the TTGO module commands the H-bridge by adjusting the pulse width modulation to control the direction and speed of the electric lifting jack. An electric lifting jack and a digital caliper are combined to form a sensing and lifting mechanism. When the user wants to lift the heel of the measurement platform to a specified height, the TTGO module in the control board reads the value of the digital caliper through an inter-integrated circuit and then performs feedback control of the electric lifting jack to lift it to the specified height. The power of the entire system is supplied by a 12-V 7800 mAh lithium battery module. The battery module comprises 12 3.65-V 2600-mAh lithium batteries (18650, Shenzhen Baiguan Battery Co., Ltd., Shenzhen, China) in series. The battery module supplies power to the 5-V control board, 12-V electric lifting jack, and 42 3.3-V FSR sensors. Voltage conversion is performed by the step-down power module in the control board.

### 2.3. Variable Collection

In addition to personal foot feature data, this study recorded the maximal safe range for heel height and other data from the FSR sensors. We referred to past research papers [17,18,19,20] on the collection of plantar pressure, and followed their methods and procedures to collect cases. Participants stood on the measurement platform (Figure 1) such that the physician could estimate the critical heel height for shoes. After the physicians estimated the maximum safe heel height, the participants’ data were collected using the FSR sensors.

#### 2.3.1. Evaluation of Maximal Safe Range of Heel Height

**Figure 2:**
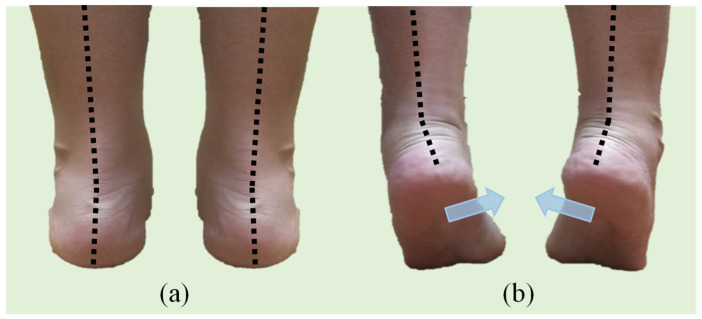
(**a**) Standing naturally; (**b**) inversion of the calcaneus during elevation.

**Figure 3:**
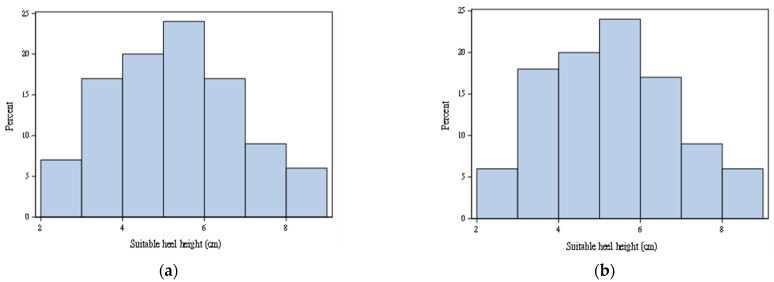
(**a**) Maximal observed safe heel height for (**a**) right foot; (**b**) left foot.

Physicians estimated the maximal safe height of high-heeled shoes by diagnosing whether a participant’s calcaneus was inverse (as Figure 2) during the continuous elevation of the heels by using the measurement platform as illustrated in Figure 1. The entire diagnosis process was repeated three times to avoid misjudgments and averaged to reduce errors. Figure 3 provides the histogram of the maximal observed safe heel height for right and left feet. The data distributions for the right and left feet were similar. The range of these samples is between 2 to 9 cm. Among 100 participants, maximal safe heights for 18 participants were different. Two participants had the largest difference, which was 0.5 cm.

#### 2.3.2. Plantar Pressure

**Figure 4:**
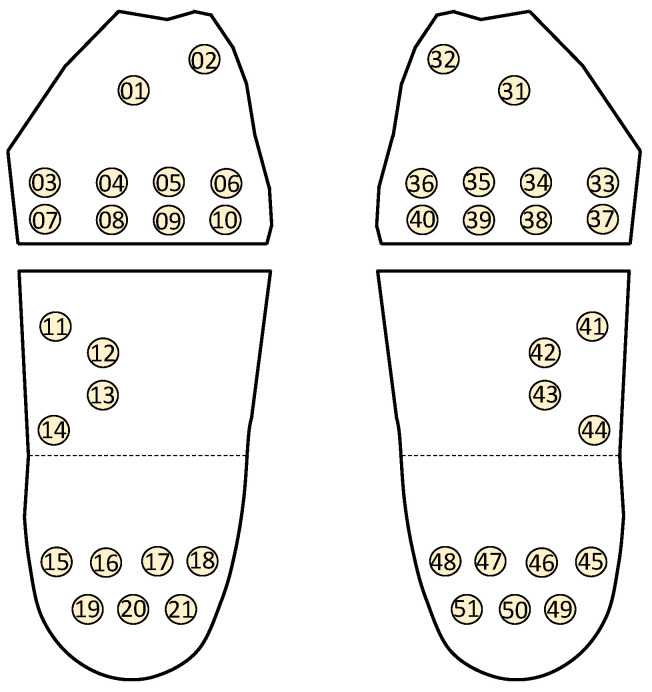
Sensor positions for left and right feet.

To measure the force exerted on different areas of the foot while standing, 42 FSR sensors distributed between the feet were placed on the measurement platform. The distribution of foot pressure in a normal foot shows a shape similar to a beautiful question mark, and the pressure is evenly distributed, extending outward from the medial toes of the forefoot, towards the lateral side of the foot and connecting to the rear heel. Thus, we can place the FSR in these areas where there will be more foot pressure [21,22]. Figure 4 shows the arrangement of these sensors. The participant was asked to stand on the measurement platform to collect plantar pressure data for a heel height of 0 cm. Then, the electric lifting jack was lifted in steps of 0.5 up to 10 cm to collect the corresponding plantar pressure data. In this manner, 21 sets of plantar pressure data for each foot were collected for each participant.

Sensors FSR01, FSR03, FSR12, FSR15, FSR19, and FSR 21 for the left foot did not provide sufficient data for use in the analysis. Except for FSR01, 97% of the plantar data was zeros in the left feet. About 15% of plantar data for FSR01 was available, but plantar data for the lower heights were not available. Thus, FSR01 was also removed. Furthermore, sensors FSR11 and FSR14 for the left foot had a relatively higher percentage of 0 and were treated as a dichotomized variable with 1 for plantar pressure larger than 0 and 0 otherwise. Finally, again owing to the data availability, sensors FSR41–FSR43, and FSR45 for the right foot were removed. In turn, 15 FSRs and 19 FSRs were used afterwards.

### 2.4. Data Analysis

**Figure 5:**
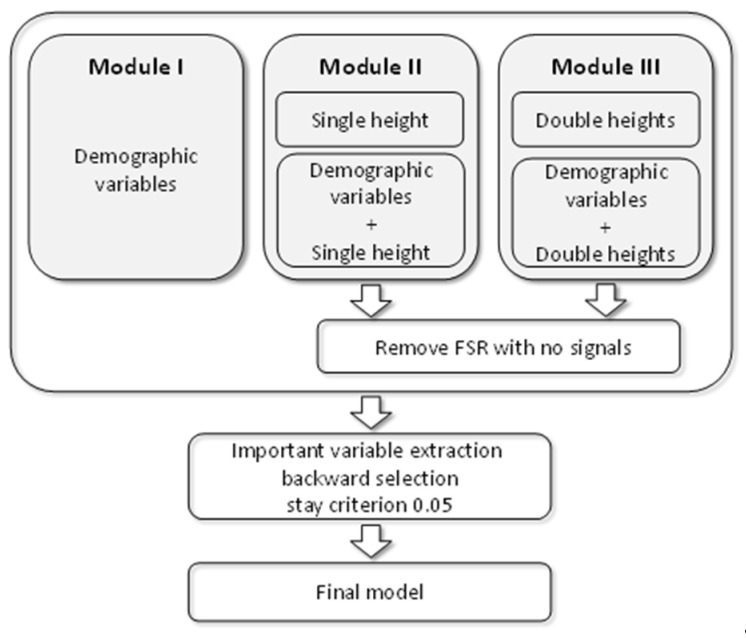
Flowchart for selecting final model for modules.

To differentiate the plantar pressure for each measured height (H), the deviation of the observed maximal safe heel height (OHH) and the height for a given plantar pressure was used as the outcome (DHH) defined as *DHH* = *OHH* − *H*. Three modules were considered in this study, as shown in Figure 5. The regression model was used to build a model for predicting *DHH* [23,24]. Let the predicted value obtained from the regression model be denoted as *pHH*. The final predicted maximal safe heel height (SHH) was then obtained as:

(1) SHH=pHH+H,

where *H* equals 0, 0.5, …, 10 cm. *SHH*, *H*, and *OHH* are all measured in centimeters (i.e., unit: cm). Because the data were collected for the left and right feet separately, the model was established for each foot.

**Figure 6:**
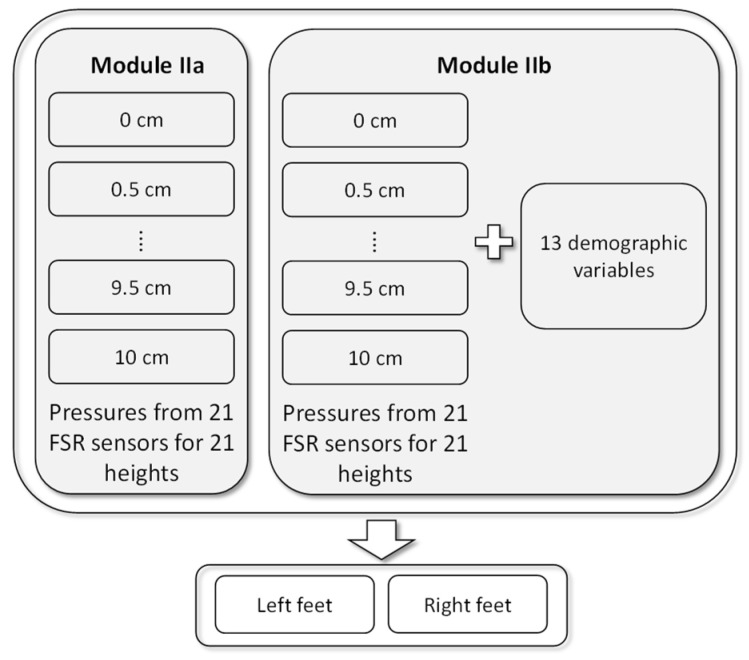
Models in Module II.

Module I used only personal features to build the model. Module II included two submodules. Module IIa used the plantar pressures collected from the FSR sensors to build the regression model. Because the plantar pressures were collected from 21 heights, the regression model was used to predict the DHH for each height. Module IIb used the plantar pressure data from the FSR sensors as well as the personal features to build the model. Figure 6 shows the structure of Module II.

**Figure 7:**
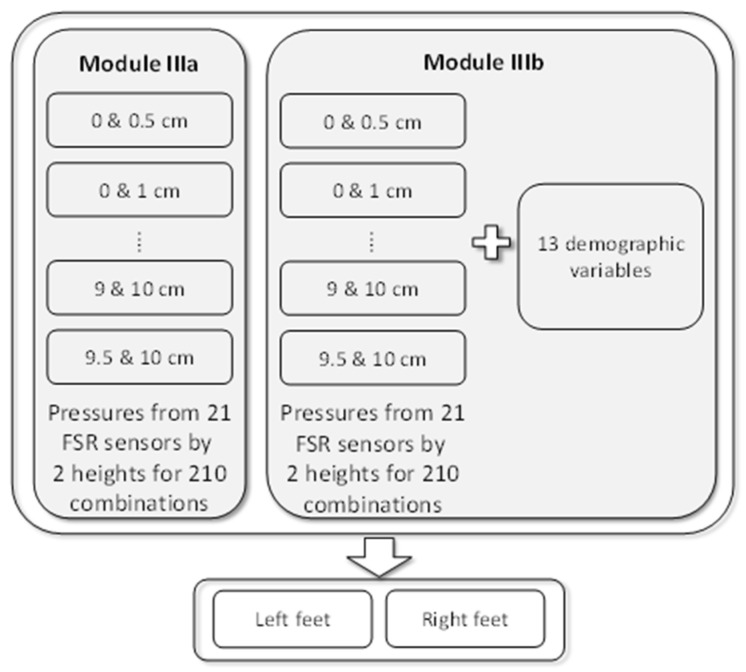
Models in Module III.

Module III used the plantar pressure data from two heights to measure the change in plantar pressure. When two heights among the 21 heights were selected, 210 different combinations are possible. For each combination, Module IIIa used the plantar pressures collected from the FSR sensors for the two heights to build the regression model. Based on the same structure as Module IIIa, Module IIIb additionally used personal features to build the model. Figure 7 shows the structure of Module III.

The coefficient of determination (*R*^2^) was used to assess the predictive power of each model. A value closer to 1 meant that the model had excellent predictive power [25]. Because Module III had two measured heights for the plantar pressures, the final predicted heel height for this module was defined as the average of the two predicted maximal safe heel heights. The overall average of mean absolute error (MAE), defined as the average of the absolute value of the predicted maximal safe heel height minus the maximal safe height determined by the physician was also used to evaluate the prediction error. SAS 9.4 (SAS Institute Inc., Cary, NC, USA) was used to conduct data analysis [26].

## 3. Results

### 3.1. Evaluation Results Based on Personal Features

Table 1 lists the summary statistics for the personal features. The participants’ average age, height, and weight were 21 years, 160 cm, and 52 kg, respectively. The basic characteristics for the left and the right feet were similar. The average foot length, forefoot width, foot height, and foot step were 24, 9.4, 5, and 22 cm, respectively. The average most convex point of the navicular bone (MCP-NB) and arch rate were 3 cm and 12.56%. The average HVA and QVA were roughly 12° and 10°, respectively, and those for the left foot were slightly larger than those for the right foot. The average rearfoot width was 6.3 cm. The average angles of the rearfoot for the left and the right foot differed slightly, being 0.89° and 0.80°, respectively. Because this study only included healthy female participants, the variation in the CVA of the rearfoot was small. This variable was categorized into three groups according to the CVA of the rearfoot: 0°, 1°, and >1°. By using 0° as the reference group, two dummy variables CVA1 and CVA2 were created for establishing the model.

Module I used all personal features to build the model shown in Table 2. By using 0.05 as the selection for a staying criterion, three features—height, weight, and MCP-NB—for both feet were found to be significant. Both height and MCP-NB were positively associated with DHH, and weight was negatively associated. For the right foot, MCP-NB was the most significant predictor for the DHH, followed by the height. By contrast, for the left foot, height was the most significant predictor, followed by MCP-NB. These variables explained approximately 17% and 14% of the variation for the right and left foot, respectively.

### 3.2. Evaluation Results Based on FSR Sensors from One Height

**Figure 8:**
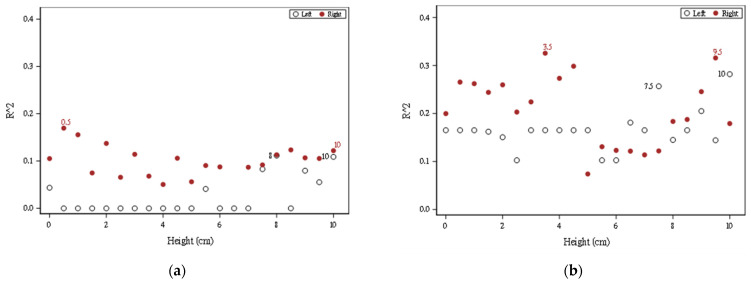
*R*^2^ values for 21 models for Module II (**a**) using plantar pressures; (**b**) using plantar pressures and personal features.

Module II used the plantar pressure data collected from FSR sensors for 21 heights to predict the DHH. A total of 21 models were generated for each foot. When 0.05 was the selection for a staying criterion, Figure 8a shows the percentage of the variation explained by Module IIa based on the plantar pressure data, where ● and ○, respectively, denote the right and left foot and the number indicates the height for the two highest predictive powers. For the right foot, the plantar pressure data measured at all heights had some ability to predict DHH. In particular, the plantar pressure data measured at 0.5 cm provided the highest *R*^2^. For the left foot, only the plantar pressure data measured at heights of 0, 5.5, 7, 8, 9, 9.5, and 10 cm had some ability to predict DHH. The plantar pressure data measured at heights 8 and 10 offered more information to predict DHH.

For the right foot, all models had some predictive power. The highest predictive power for the model using data measured at a height of 0.5 cm was 17%. As shown in Table 3, FSR31, FSR39, and FSR49 were chosen when data measured at this height were used. FSR31 was negatively associated with DHH, whereas the other two sensors were positively associated. For the left foot, the highest predictive power for the model using data measured at heights of 8 and 10 cm was 11%. Table 3 shows the model when using data measured at 10 cm; the model when using data measured at 8 cm is not shown. FSR08 and FSR17 were significantly and negatively associated with DHH.

Figure 8b shows the percentage of variation explained by Module IIb based on the plantar pressure data and the personal features, where ● and ○, respectively, denote the right and left foot and the number indicates the height for the two highest predictive powers. The predictive power increased gradually. For the right foot, the highest predictive powers for the model using data measured at 3.5 and 9.5 cm were 34% and 32%, respectively. By contrast, for the left foot, the highest predictive powers for the model using data measured at 7.5 and 10 cm were 26% and 28%, respectively.

Table 4 shows the significant predictors for the model using data measured at 3.5 cm for the right foot. Four FSR sensors—FSR34, FSR39, FSR48, and FSR50—were significantly and positively associated with DHH and FSR33 was negatively associated. The personal features including height, weight, MCP-NB, CVA1, CVA2, and forefoot width were found to be significantly associated with DHH, and of these, only weight was negatively associated. For the model using data measured at 3.5 cm, the most significant predictors were weight, FSR34, and height.

Table 4 shows the model estimates obtained using measurements at 10 cm for the left foot. FSR08 and FSR17 were significantly and negatively associated with DHH. Personal features including height, weight, MCP-NB, CVA1, and CVA2 were also significantly associated with DHH, whereas only weight was negatively associated.

### 3.3. Evaluation Results Based on FSR Sensors from Two Heights

**Figure 9:**
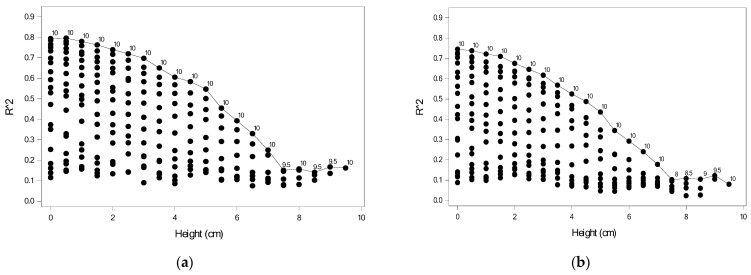
*R*^2^ values based on plantar pressures for 210 models for Module IIIa: (**a**) right foot; (**b**) left foot.

Module III was established using sensor data collected from two heights. Selecting two heights from among 21 heights resulted in 210 possible combinations, and thus, 210 models were established for each foot. Figure 9 shows the predictive power of Module IIIa for the 210 models for the right and left feet, where each point indicates *R*^2^ for one combination. For a given height, the number indicates the second height for the highest *R*^2^. When using data collected from two extreme heights, the model would yield the highest predictive power. For example, for the right foot as shown in Figure 9a, the models using data measured at 0.5 and 10 cm had the highest predictive power (*R*^2^ = 0.806), followed by the model using data measured at 0 and 10 cm (*R*^2^ = 0.796). Figure 9b displayed the result for the left foot and the models using data measured at 0 and 10 cm had the highest predictive power (*R*^2^ = 0.746), followed by the model using data measured at 0.5 and 10 cm (*R*^2^ = 0.738).

Table 5 shows the model estimates for the model with the highest predictive power for the right foot. Nine FSR sensors were significantly associated with DHH; specifically, FSR32, FSR34, FSR35, FSR36, and FSR50 were negatively associated, whereas FSR39, FSR 44, FSR47, and FSR51 were positively associated. Table 5 also shows the model estimates for the model with the highest predictive power for the left foot. Eight FSR sensors were significantly associated with DHH; specifically, FSR04, FSR05, FSR06, and FSR20 were negatively associated, whereas FSR08, FSR11, FSR13, and FSR17 were positively associated.

**Figure 10:**
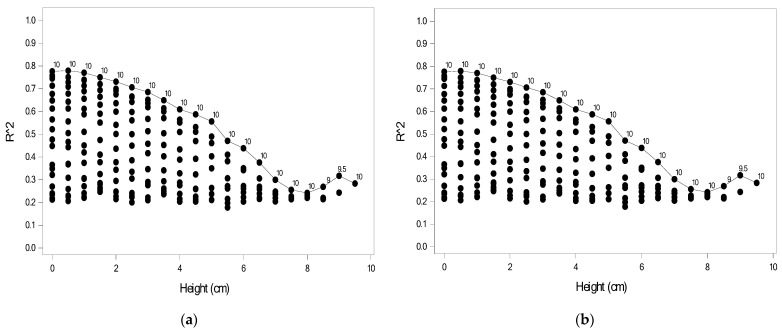
*R*^2^ based on plantar pressures and personal features for 210 models for Module IIIb: (**a**) right foot; (**b**) left foot.

In addition to the FSR sensor data, 210 models including the personal features for each foot were built. Figure 10 shows the predictive power of Module IIIb for 210 models for the right and left foot, where each point indicates *R*^2^ for one combination. When the personal features were added, the predictive power increased. The model with the highest predictive power was derived from the sensor data measured at two extreme heights, as shown in Figure 10. For the right foot, the models using data measured at 0.5 and 10 cm had the highest predictive power (*R*^2^ = 0.820), followed by the model using data measured at 0 and 10 cm (*R*^2^ = 0.817). For the left foot, the models using data measured at 0.5 and 10 cm had the highest predictive power (*R*^2^ = 0.779), followed by the model using data measured at 0 and 10 cm (*R*^2^ = 0.777). The predictive power with the combination of 0 and 10 cm was similar to that with the combination of 0.5 and 10 cm. However, the combination of 0 and 10 cm avoids the need for raising the measurement platform one time, making it more convenient in practice.

Table 6 shows the estimates for the model using data measured at heights of 0.5 and 10 cm for the right foot. FSR32, FSR33, FSR34, FSR35, FSR36, and FSR50 were negatively associated with DHH, whereas FSR39, FSR40, FSR44, FSR47, and FSR51were positively associated. Furthermore, MCP-NB and HVA were positively associated with DHH. The fitted model for the right foot was given as (2).

*pHH* = −7.27 − 1.47 FSR32 − 5.81 FSR33 − 2.55 FSR34 − 2.30 FSR35 − 5.28 FSR36 + 3.52 FSR39 + 3.58 FSR40 + 11.16 FSR44 + 8.10 FSR47 − 10.4 FSR50 + 4.59 FSR51 + 2.24 MCP-NB + 0.09 HVA (2)

Table 6 also shows the model estimates for the model using data measured at heights of 0 and 10 cm for the left foot. FSR02, FSR04, FSR05, FSR06, and FSR20 were negatively associated with DHH, whereas FSR11, FSR13, and FSR17 were positively associated. Additionally, height, CVA1, and CVA2 were positively associated with DHH. Overall, both models selected the same significant variables and had similar estimates. The fitted model for the left foot was given as (3).

*pHH* = −24.76 − 4.65 FSR02 − 3.17 FSR04 − 5.02 FSR05 − 4.18 FSR06 + 1.56 FSR11 + 14.88 FSR13 + 7.87 FSR17 − 3.37 FSR20 + 0.16 height + 1.10 CVA1 + 1.32 CVA2 (3)

Table 5 and Table 6 show the most important variables for the left and right feet individually, including the most important FSR numbers. We compared the more important FSRs in Table 5 and Table 6 according to the front, middle, and back positions of the soles of the feet, and organize them into Table 7.

If we want to know which personal features besides FSRs have better predictive ability for the height of high heels, we can organize and compare the data in Table 2 and Table 6, which can be organized into Table 8.

## 4. Discussion

The model (Module IIIb) shown in Table 6 was used to compute the predicted DHH. The predicted heel height was derived from the measured height of the plantar pressure and DHH, as shown in (1). Because the model for the right foot used the plantar pressure measured at 0.5 and 10 cm for the right foot, the predicted heel height was computed by averaging that measured at 0.5 and 10 cm, respectively. For the left foot, the plantar pressure was measured at 0 and 10 cm. The corresponding MAE of the model for the right and left feet was 1.22 and 1.57 cm, respectively. The overall MAE was 1.40 cm, which was obtained by averaging the MAE of the right and left feet; this was slightly higher than the value of 1.21 cm obtained using the system proposed by Lee et al. [12]. Lee et al. noted that heel heights are normally classified into low-heeled shoes (<2.54 cm), mid-heeled shoes (2.54–6.35 cm), and high-heeled shoes (>6.35 cm). The proposed model had an MAE of less than half of an inch, indicating that it has an excellent ability to distinguish these classes. However, the method used in the study by Lee et al. requires the user to continuously shoot 21 times through the cameras, which takes about 12 min. At the same time, an expensive computer with a graphics processing unit is required to perform AI calculations. Compared with the equipment and AI model proposed in [12], by measuring the plantar pressure at 0 and 10 cm and using some personal features, the proposed model was easier to implement and cost less time (about 2 min).

When using the plantar pressure data from two heights, the model performance in terms of *R*^2^ improved substantially. In particular, the model based on the plantar pressure data measured at two extreme heights had the highest predictive power. Table 7 shows a comparison of the positions of the significant sensors for Modules IIIa and IIIb. For the model for the right foot, eight sensors were consistently found. Increasing the plantar pressure for sensors FSR32, FSR34, FSR35, and FSR36 in the front and sensor FSR50 in the back reduced SHH, whereas increasing the plantar pressure for sensor FSR39 in the front, sensors FSR44 in the middle, and FSR47 and FSR51 in the back increased SHH. For the model for the left foot, six sensors were the same and three sensors were different. Increasing the plantar pressure for sensors FSR04 and FSR05 in the front and sensor FSR20 in the back reduced DHH, whereas increasing the plantar pressure for sensors FSR11 and FSR13 in the middle, and FSR17 in the back increased SHH. The inconsistent sensors were all found in the front for both feet. Because the positions of the sensors were fixed on the platform, they could not be adjusted according to foot size. This might have resulted in inconsistencies.

Table 8 shows comparisons of the significant personal features from Modules I and IIIb. MCP-NB was an important variable for all models. For the right foot, weight was significant for Module I and HVA was significant for Module IIIb. For the left foot, height was significant in both models. CVA1 and CVA2 were only significant for Module IIIb.

As can be seen in Figure 9 and Figure 10, the smaller the difference of DHH, the smaller its *R*^2^, regardless of the Module IIIa or IIIb and the left or right feet. This is also in line with the theory of other foot research [9,10], that the higher the heel, the greater the pressure on the toe becomes, and the less the pressure on the heel becomes. That is, the greater the “pressure difference” between the toe and the heel, the better the prediction effect.

The plantar pressure data collected from the left and right foot seemed to differ. Participants tended to put more weight on their dominant foot while standing on the platform. This might be the reason why our sample had more data on the right feet. This might induce the fitted model for the right foot to have better predictive power. For future study, the subject is advised to stand straight so that the plantar pressure can be obtained for both feet. Owing to the limited data, the important FSRs were not investigated. It might be possible to use fewer FSRs such that FSR can be positioned slightly closer to the center to have a robust system with respect to the foot size.

## 5. Conclusions

This study proposed a novel approach to assess the tolerable heel height of high-heeled shoes for female participants by using FSR sensor data with regression models. In this study, three regression models—Modules I, II, and III—were tested and compared. After experimentation, Module IIIb was found to have the best predictive ability. Furthermore, the right foot had better predictive ability than the left foot. The model of Module IIIb included data collected from plantar pressures measured at two extreme heights and personal features, and it had optimal predictive power. Personal features may not always be easy to obtain. Thus, for practical usage, the model of Module IIIa that includes only plantar pressures measured at two extreme heights for both feet is recommended. The proposed model can predict the heel height and provide maximal safe guidance for women to understand their limitations in wearing high-heeled shoes without consulting physicians. When the models for both feet provided different maximal safe heights, the lowest one should be considered. In the future, these lightweight, inexpensive, and convenient FSR sensors could be incorporated into insoles for regular use through the smartphone APP.

## Figures and Tables

**Table 1:** Descriptive statistics for demographics.

	Right		Left	
Variable	Mean	SD	Mean	SD
Age			21.12	1.25
Height (cm)			159.9	5.43
Weight (kg)			51.81	6.38
Foot length (cm)	24.08	1.04	24.00	1.04
Forefoot width (cm)	9.41	0.57	9.40	0.58
Foot circumduction (cm)	22.36	1.05	22.29	1.08
Most convex point of the navicular bone (MCP-NB)	3.03	0.25	3.03	0.23
Arch rate	12.55	0.89	12.58	0.84
Hallux valgus angle (HVA)	12.11	4.36	12.61	3.91
Quintus varus angle (QVA)	10.26	4.10	10.56	3.87
Calcaneus varus angle (CVA)				
CVA = 0 degree	46	46%	47	47%
CVA = 1 degree	34	34%	27	27%
CVA > 1 degree	20	20%	26	26%
Rearfoot width (cm)	6.34	0.36	6.31	0.34
Heel height (cm)	5.08	1.59	5.08	1.58

**Table 2:** Regression models based on personal features.

	Right			Left		
Variable	Est	SE	*p*	Est	SE	*p*
Intercept	−12.3	4.74	0.0112	−10.8	4.79	0.0262
Height	0.09	0.03	0.0126	0.09	0.03	0.0155
Weight	−0.06	0.03	0.0431	−0.06	0.03	0.0485
MCP-NB	2.15	0.73	0.0038	1.79	0.73	0.0163
*R* ^2^	0.17			0.14		

**Table 3:** Regression models based on one plantar height.

	Right			Left		
Variable	Est	SE	*p*	Est	SE	*p*
Intercept	4.05	0.21	<0.001	−4.27	0.24	<0.001
FSR08				−2.45	1.10	0.029
FSR17				−5.02	1.61	0.002
FSR31	−10.5	4.54	0.023			
FSR39	7.32	2.54	0.005			
FSR49	4.90	1.55	0.002			
*R* ^2^	0.17			0.11		

**Table 4:** Regression models based on plantar pressure for one height and personal features.

	Right			Left		
Variable	Est	SE	*p*	Est	SE	*p*
Intercept	−19.8	4.81	<0.0001	−19.8	4.93	0.0001
FSR08				−2.39	1.03	0.0228
FSR17				−3.49	1.70	0.0426
FSR33	−9.90	3.82	0.0112			
FSR34	4.63	1.52	0.0032			
FSR39	3.06	1.54	0.0494			
FSR48	11.68	5.78	0.0465			
FSR50	2.58	1.11	0.0218			
Height	0.10	0.03	0.0062	0.07	0.03	0.0286
Weight	−0.11	0.03	0.0007	−0.06	0.03	0.0473
MCP-NB	1.73	0.69	0.0135	1.99	0.71	0.0064
CVA1	0.48	0.32	0.1389	0.73	0.34	0.0381
CVA2	0.94	0.38	0.0151	0.70	0.34	0.0453
Forefoot width	0.85	0.46	0.0689			
*R* ^2^	0.34			0.28		

**Table 5:** Regression models based on plantar pressure for two heights.

	Right			Left		
Variable	Est	SE	*p*	Est	SE	*p*
Intercept	1.01	0.49	0.0391	0.39	0.67	0.5601
FSR04				−3.30	1.25	0.0088
FSR05				−4.87	0.76	<0.0001
FSR06				−3.74	1.47	0.0119
FSR08				3.79	1.64	0.0218
FSR11				1.59	0.61	0.0098
FSR13				14.81	6.23	0.0185
FSR17				7.55	1.23	<0.0001
FSR20				−2.13	0.90	0.0191
FSR32	−1.13	0.39	0.0043			
FSR34	−2.90	0.78	0.0003			
FSR35	−2.38	0.45	<0.0001			
FSR36	−4.97	1.11	<0.0001			
FSR39	5.67	1.58	0.0004			
FSR44	12.59	2.24	<0.0001			
FSR47	7.81	1.47	<0.0001			
FSR50	−10.2	1.80	<0.0001			
FSR51	4.86	1.29	0.0002			
*R* ^2^	0.80			0.75		

**Table 6:** Regression models based on plantar pressure for two heights and personal features.

	Right			Left		
Variable	Est	SE	*p*	Est	SE	*p*
Intercept	−7.27	2.16	0.0009	−24.6	5.97	<0.0001
FSR02				−4.65	1.80	0.0105
FSR04				−3.17	1.14	0.0060
FSR05				−5.02	0.72	<0.0001
FSR06				−4.18	1.42	0.0036
FSR11				1.56	0.56	0.0061
FSR13				14.88	5.92	0.0129
FSR17				7.87	1.23	<0.0001
FSR20				−3.37	0.92	0.0003
FSR32	−1.47	0.39	0.0002			
FSR33	−5.81	2.64	0.0287			
FSR34	−2.55	0.79	0.0014			
FSR35	−2.30	0.44	<0.0001			
FSR36	−5.28	1.09	<0.0001			
FSR39	3.52	1.59	0.0278			
FSR40	3.58	1.60	0.0264			
FSR44	11.16	2.17	<0.0001			
FSR47	8.10	1.41	<0.0001			
FSR50	−10.4	1.71	<0.0001			
FSR51	4.59	1.23	0.0002			
Height				0.16	0.04	<0.0001
MCP-NB	2.44	0.68	0.0004			
HVA	0.09	0.04	0.0146			
CVA1				1.10	0.45	0.0143
CVA2				1.32	0.46	0.0043
*R* ^2^	0.82			0.78		

**Table 7:** Comparison of sensor positions for Modules IIIa and IIIb.

Type of Association		Right Foot			Left Foot		
		Front	Middle	Back	Front	Middle	Back
Positive	Consistent	39	44	47, 51		11, 13	17
	Inconsistent	40 #			8 *		
Negative	Consistent	32, 34, 35, 36		50	4, 5		20
	Inconsistent	33 #			2 #, 6		

* Only derived from Module IIIa; # only derived from Module IIIb.

**Table 8:** Comparisons of personal features for Modules I and IIIb.

	Right Foot		Left Foot	
	Module I	Module IIIb	Module I	Module IIIb
Height	✓		✓	✓
Weight	✓		✓	
HVA		✓		
MCP-NB	✓	✓	✓	✓
CVA1 and CVA2				✓

## Data Availability

Not applicable.
